# ICTV Virus Taxonomy Profile: Mymonaviridae 2022

**DOI:** 10.1099/jgv.0.001787

**Published:** 2022-11-18

**Authors:** Dàohóng Jiāng (姜道宏), María A. Ayllón, Shin-Yi L. Marzano, Hideki Kondō (近藤秀樹), Massimo Turina, ICTV Report Consortium

**Affiliations:** 1 Huazhong Agricultural University, Wuhan, PR China; 2 Centro de Biotecnología y Genómica de Plantas (UPM-INIA) and Dpto. Biotecnología-Biología Vegetal, ETSIAAB, Universidad Politécnica de Madrid, Madrid, Spain; 3 United States Department of Agriculture, Agricultural Research Service, Toledo, Ohio, USA; 4 Institute of Plant Science and Resources (IPSR), Okayama University, Okayama, Japan; 5 Institute for Sustainable Plant Protection, Torino 10135, Italy

**Keywords:** *Mymonaviridae*, mymonavirid, mymonavirus, *Mononegavirales*, taxonomy, classification, ICTV Report

## Abstract

Typical members of the family *Mymonaviridae* produce filamentous, enveloped virions containing a single molecule of linear, negative-sense RNA of about about 10 kb, but some may not produce any virions. The family includes several genera, some with multiple species. Mymonavirids usually infect filamentous fungi, but a few have been identified associated with insects, oomycetes or plants. At least one virus, Sclerotinia sclerotiorum negative-stranded RNA virus 1, induces hypovirulence in its fungal host. This is a summary of the International Committee on Taxonomy of Viruses (ICTV) Report on the family *Mymonaviridae*, which is available at ictv.global/report/mymonaviridae.

## Virion

**Fig. 1.**
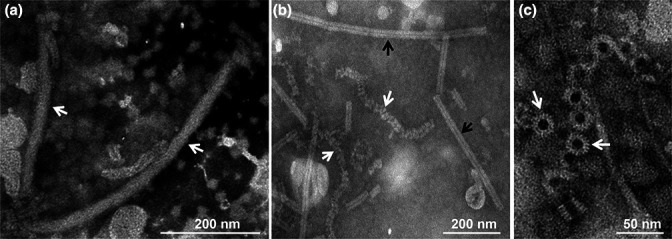
Morphology of nucleoprotein–RNA complexes (RNPs) of SsNSRV-1. (**a**) Filamentous, enveloped virions (white arrows), (**b**) purified tight (black arrows) or loose (white arrows) coils of RNP complexes, and (**c**) rings that constitute the coils and nucleoprotein (NP) monomers (modified from [1]).

Virions of a typical member, Sclerotinia sclerotiorum negative-stranded RNA virus 1 (SsNSRV-1), are filamentous, 25–50 nm in diameter, about 1000 nm in length and enveloped by a membrane (Table 1). The outer surface of virions does not appear to be covered with spikes. The nucleocapsids released from virions are single, left-handed, helical structures that, when tightly coiled, have a diameter of 20–22 nm and a length of 200–2000 nm. Nucleocapsids consist of polymerized nucleoprotein (NP) monomers (Fig. 1). Whether other members are able to produce virions has not been investigated.

**Table 1.** Characteristics of members of the family *Mymonaviridae*

Example:	Sclerotinia sclerotiorum negative-stranded RNA virus 1 (KJ186782), species *Sclerotimonavirus sclerotinae*, genus *Sclerotimonavirus*
Virion	Enveloped, filamentous virions 25–50 nm in diameter and about 1000 nm in length
Genome	Single molecule of linear, negative-sense RNA of about 10 kb
Replication	Ribonucleoprotein (RNP) complexes containing anti-genomic RNA serve as templates for synthesis of nascent RNP complexes containing genomic RNA
Translation	The virus RNA-directed RNA polymerase binds the encapsidated genome at the leader region and then sequentially transcribes each gene by recognizing start and stop signals flanking viral genes. This produces subgenomic RNAs that serve as mRNAs
Host range	Fungi; also detected in metagenomic studies of insects, oomycetes and plants
Taxonomy	Realm *Riboviria,* phylum *Negarnaviricota*, subphylum *Haploviricotina*, class *Monjiviricetes*, order *Mononegavirales*; the family includes >8 genera, at least six of which include multiple species

## Genome

**Fig. 2.**
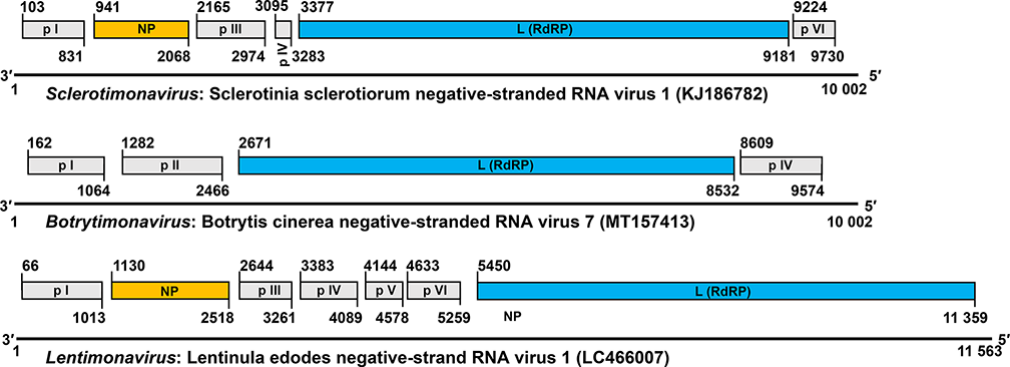
Genome organization of members of the genera *Sclerotimonavirus* [1], *Botrytimonavirus* [2] and *Lentimonavirus* [3]. The position of each ORF is indicated above the negative-sense strand. ORFs encoding putative proteins with unknown function are indicated in grey. NP, nucleoprotein; RdRP, RNA-directed RNA polymerase.

Virions of SsNSRV-1, the best-studied mymonavirus, contain a single molecule of a 10 002 nt, linear, negative-sense RNA genome lacking a poly(A) tail at the 3′-terminus and uncapped at the 5′-terminus. The two termini are not complementary in sequence. This genome is predicted to have six major non-overlapping ORFs (encoding proteins p I, NP, p Ⅲ, p Ⅳ, L protein and p Ⅵ) expressed as individual transcription units and separated by non-coding intergenic regions containing highly conserved gene junction sequences (Fig. 2). The nucleoprotein encapsidates the mymonavirus genome. The RNA-directed RNA polymerase, part of the large protein (L), mediates virus genome replication and transcription. The functions of the remaining four proteins are unclear. The genomes of other mymonaviruses range from 6.2 to 11.6 kb with four to seven ORFs, although some genomes may be incomplete (Fig. 2).

## Replication

Mymonavirids are believed to replicate in the cytoplasm of host cells, but their replication strategies are not well studied. Ribonucleoprotein (RNP) complexes can be used directly as templates for replication and transcription. Replication usually occurs on RNP complexes and requires L protein to synthesize full-length positive-sense antigenomes that serve as templates for the synthesis of negative-sense progeny genomes.

## Pathogenicity

Mymonavirids have been characterized in fungi [1,4] and in samples of insects [5,6], oomycetes [7], plants [8,9] and soil [10] by high-throughput sequencing; their real hosts are unclear. SsNSRV-1, a typical mymonavirid infecting *Sclerotinia sclerotiorum*, causes its host to grow slowly and lose its pathogenicity. SsNSRV-1 can be transmitted horizontally through hyphal fusion and be transfected by using virions and host protoplasts [1].

## Taxonomy

Current taxonomy: ictv.global/taxonomy. Mymonavirids form a family in the haploviricotine order *Mononegavirales*. Within this order, mymonavirids are most closely related to members of the families *Bornaviridae*, *Lispiviridae*, *Nyamiviridae* and *Rhabdoviridae*.

## Resources

Full ICTV Report on the family *Mymonaviridae*: ictv.global/report/mymonaviridae.

## Abbreviations

L: large protein
NP: nucleoprotein
RdRP: RNA-directed RNA polymerase
RNP: ribonucleoprotein
SsNSRV-1: Sclerotinia sclerotiorum negative-stranded RNA virus 1
